# Hearing loss: a global view for gene therapy approaches and challenges

**DOI:** 10.1007/s00431-025-06426-9

**Published:** 2025-08-27

**Authors:** Nagham Maher Elbagoury

**Affiliations:** Medical Molecular Genetics Department, Human Genetics and Genome Research Institute, National Research Centre, Cairo, 12311 Egypt

**Keywords:** Hearing loss, AAV, Delivery routes, Gene therapy, Challenges

## Abstract

Hearing loss (HL) is a global issue that affects the quality of life. The incidence of the prelingual form reaches 1: 500 live births, where inherited HL represents the most frequent form. The early diagnosis and intervention to restore hearing might help in reaching near-normal speech milestones in congenital HL cases. Current treatment includes using a hearing aid and cochlear implantation (CI). In spite of the improvement detected on using these treatments, hearing is never back to normal. Gene therapy is the most recent approach studied in an attempt to reach a radical cure for this health problem. The tremendous genetic heterogeneity beyond different types of HL renders gene therapy a challenging yet promising treatment approach. This review encompasses all aspects of this approach including the various strategies (gene-dependent and gene-independent approaches), vehicles used for delivery, routes of administration, drawbacks, and the challenges that remain to be addressed.

## Introduction

**Fig. 1:**
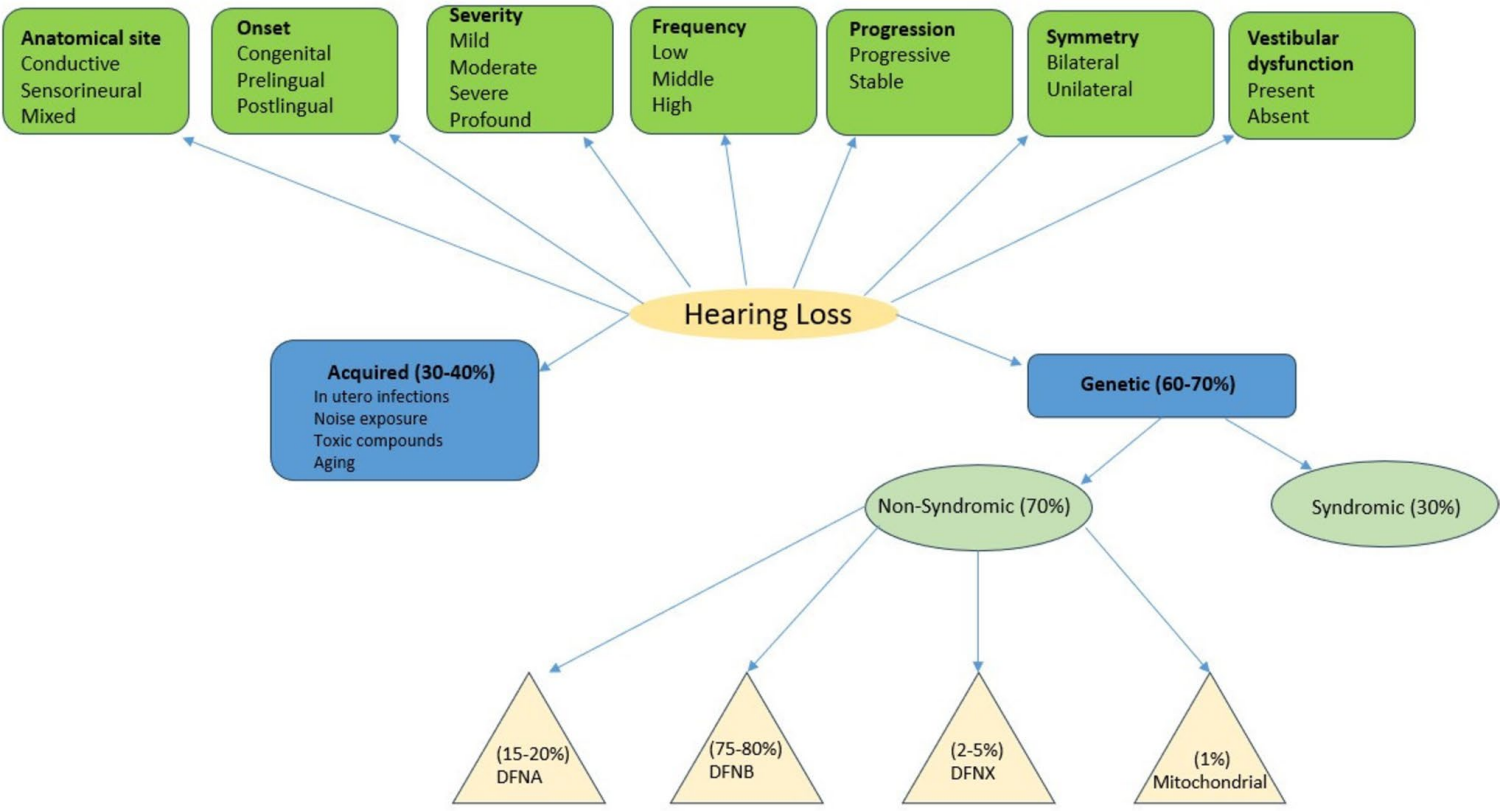
Diagram showing classifications of hearing loss. DFNA: dominant form, DFNB: recessive form, DFNX: X-linked form

Hearing Loss has a high incidence among human sensory disorders [1–3], with the prelingual form affects approximately 1 in 500 individual [4]. The World Health Organization expects the number of affected individuals to reach 2.5 billion within the next 25 years. Inherited HL represents more than half of the congenital cases [5–7]. Classification of hearing loss (HL) can follow different descriptive perspectives according to anatomical site, onset, severity, progression, symmetry, vestibular affection, perception of sound frequency, and underlying cause, whether acquired due to different environmental stresses or genetic, as shown in Fig. 1.

Radical cure is still not applicable in HL as available solutions are mainly using hearing aid devices and cochlear implantation (CI) surgery with a clinical output of subnormal tone recognition, difficulty in processing intonation, and reduced ability to appreciate music[4, 8]. In the past few years, many studies targeting gene therapy have been launched. Gene therapy is a broad topic with many different strategies underneath. The choice of which strategy to use is dependent on the affected gene, its mode of inheritance, and sometimes the pathogenic variant. In this context, this review is mainly concerned with searching the literature for updates in different hearing loss gene therapy approaches. By the end of this review, we aim to reach a clearer view of the hearing loss genetics with special emphasis on the recent gene therapy approaches.

## Evolution of the genetic landscape in HL

Exploring genetic causes underlying different types of hearing loss began in 1995 [9], and since then, the number of causative genes has been continuously increasing due to the rapid progress in developing genetic analysis techniques [10, 11]. The database of genes involved in hearing loss is very dynamic, where new genes are added while others are “refuted” or disapproved.

### Genes refuted and added

A study for curation of data refuted some HL genes when contradictory evidence outweighs evidence supporting them. It refuted the *GJB6* gene in correlation to ARNSHL, the *HARS* gene with correlation to Usher syndrome, and the *MYO1A* gene with correlation to ADNSHL [12]. On the other hand, several studies have been able to establish new correlations between some genes and inherited HL forms, as tabulated in Table 1.

**Table 1:** Examples of genes recently correlated to some HL disorders reflecting the dynamic genetic landscape of HL

Gene (OMIM#)	Function	Newly associated HL disorder (OMIM#)	Inheritance pattern	Year of correlation of the gene to HL	Reference
*CLRN2* (#618,988)	Maintaining the structure and function of stereocilia	Deafness, autosomal recessive 117 (#619,174)	AR	2021	[106]
*GAS2 (#602,835)*	Maintaining the structural integrity of cochlear supporting cells	Deafness, autosomal recessive 125 (#620,877)	AR	2021	[107]
*RIPOR2* (#611,410)	Maintaining the structure and orientation of stereocilia	Deafness, autosomal dominant 21(#607,017)	AD	2021	[108]
*TMTC4* (#618,203)	Maintaining the function of hair cells	Deafness, autosomal recessive 122 (#620,714)	AR	2023	[109]
*STX4* (#186,591)	Important for inner ear mechanotransduction	Deafness, autosomal recessive 123 (#620,745)	AR	2023	[110]
*PKHD1L1* (#607,843)	Maintaining the structure and function of stereocilia	Deafness, autosomal recessive 124 (#620,794)	AR	2024	[111]
*THBS1* (#188,060)	Formation and function of afferent synapse	Autosomal recessive non- syndromic HL (not yet on OMIM)	AR	2024	[112]
*IKZF2* (# 606,234)	An essential transcription factor for outer hair cells maturation and function	Autosomal dominant non-syndromic HL (not yet on OMIM)	AD	2024	[113]
*MAP3K1* (#600,982)	Development and maintenance of sensory hair cells	Autosomal recessive non- syndromic HL (not yet on OMIM)	AR	2024	[114]
*DAP3 (#602,074)*	Plays an important role in the inner ear, mainly in the function of mitoribosomes	Perrault syndrome 7 (#621,101)	AR	2025	[115]

OMIM: Online Mendelian Inheritance in Man, AR: Autosomal Recessive, AD: Autosomal Dominant

## Functions of genes involved in the hearing mechanism

**Fig. 2:**
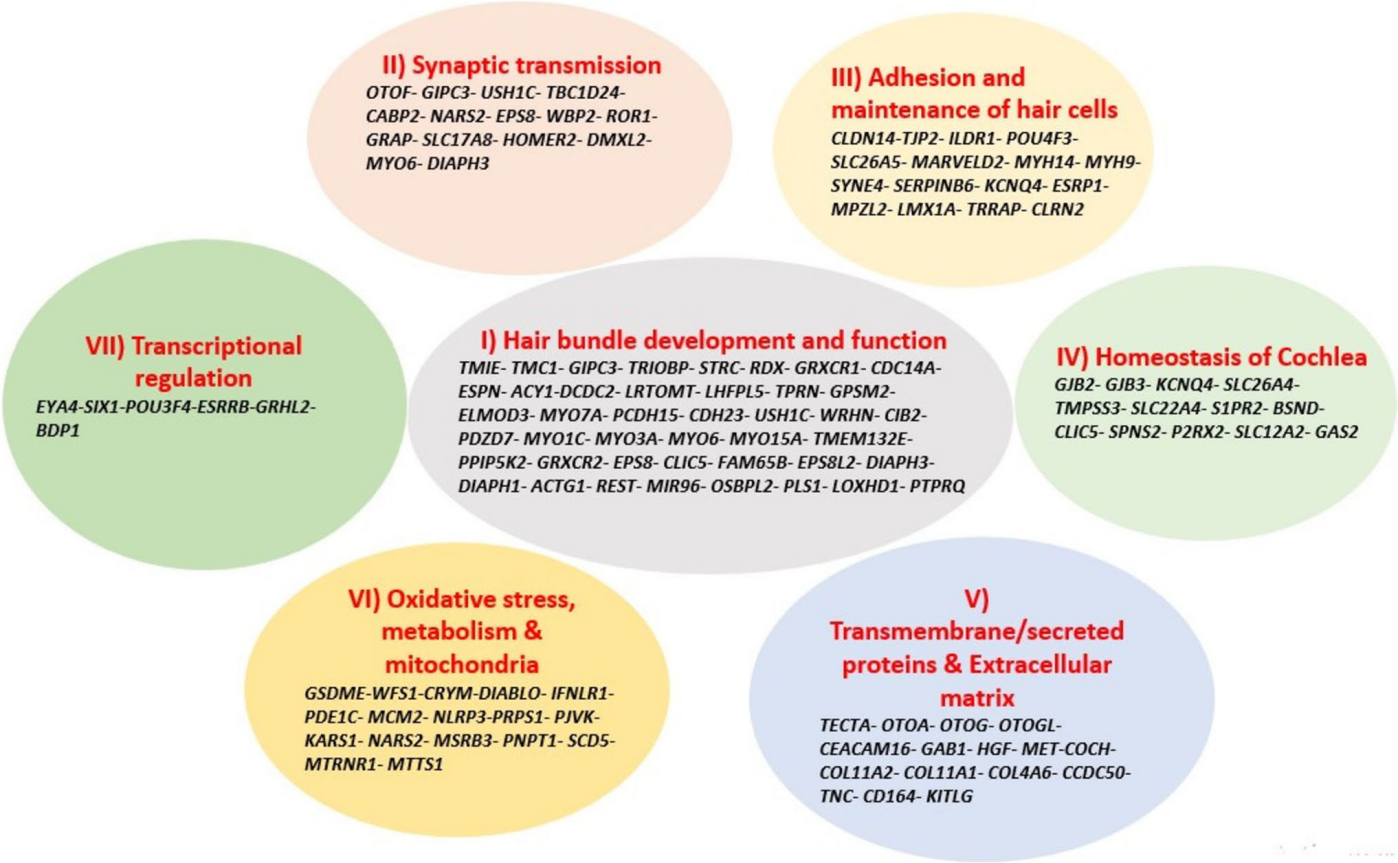
Diagram showing the different genes involved in hearing grouped according to their roles

The auditory pathway of mice resembles that of humans to a great extent. This helped in using animal models to further characterize hearing loss and predict the effect of different proteins expressed in the inner ear [4, 13, 14]. Classification of the genes on the basis of function yielded seven groups of genes, as shown in Fig. 2**.**

## Gene therapy

Building on a solid genetic background about HL helped commence the era of gene defects amending. Orchestration between delivery route, best strategy, convenient vector, and optimum therapeutic timing is a must to reach a successful result. In the upcoming section, we are going to discuss gene therapy with all approaches, strategies, used vehicles, routes of administration, and challenges.

### Vehicles

#### Viral vectors

Different viral vectors have been investigated for auditory gene delivery, namely, lentiviruses [15, 16], adenoviruses (AdVs), adeno-associated viruses (AAVs) [17, 18], and herpes simplex viruses [19, 20]. Each of them has advantages and drawbacks as shown in Table 2.

**Table 2:** Different viral and non-viral vectors

	**Transfection tool**	**Composition**	**Cargo capacity**	**Integration in host-genome**	**Transgene expression**	**Advantages**	**Disadvantages**
**Viral vector**	Adeno-associated virus	ssDNA	~ 4.8 kb	Non-integrating	Transient	Safe, replication deficiency, persistent transgene expression, high transduction efficiency	Small cargo capacity, tissue specificity, Probability of immune responses
	Adenovirus	dsDNA	7.5–36 kb	Non-integrating	Transient and permanent	Wide host range, large cargo, high transduction efficiency	Elevated immunogenicity, short transgene expression time, high pathogenicity
	Lentivirus	ssRNA	8 kb	Integrating	Transient and permanent	Wide range of host cells, large cargo capacity, reduced immunogenicity, persistent expression due to transgene integration, highly effective in dividing and nondividing cell	Elevated pathogenicity, probability of mutagenesis, replication probability, risk of virus propagation
	Herpes simplex virus	dsDNA	30 kb	Non-integrating	Transient	Large cargo capacity Neurotropic No integration	Probability of recombination, short transgene expression time, reduced transduction
**Non-viral vector**	Polymeric nanoparticles, cationic liposomes and exosomes	N/A	Variable	Non-integrating	Transient and permanent	Reduced immunogenicity, cheap, easy to prepare, large cargo capacity	Reduced transduction, decreased tissue specificity, toxicity risk
	Electroporation					Reduced immunogenicity, high transfection efficiency	Risk of tissue damage, limitation of gene transfer to targeted area, needs surgical intervention for internal organs
	Biolistic (gene gun)					Reduced immunogenicity, good in vivo activity	

ss: single-stranded, ds: double-stranded, kb: kilo-base, N/A: not available

AAVs were reported to have the least pathogenic and immunogenic effects. It has 12 serotypes with a natural capsid [21]. A lot of pseudo-serotypes have been genetically engineered to modify the capsid to adjust the cellular tropism property (ability for transduction) [22, 23].

#### Non-viral vectors

Although non-viral vectors are less efficient, they are safer for humans since they are less immunogenic and do not risk incorporation in human DNA. The different types of non-viral vectors, their cargo capacity, advantages, and disadvantages are displayed in Table 2.

### Routes of gene therapy delivery

**Fig. 3:**
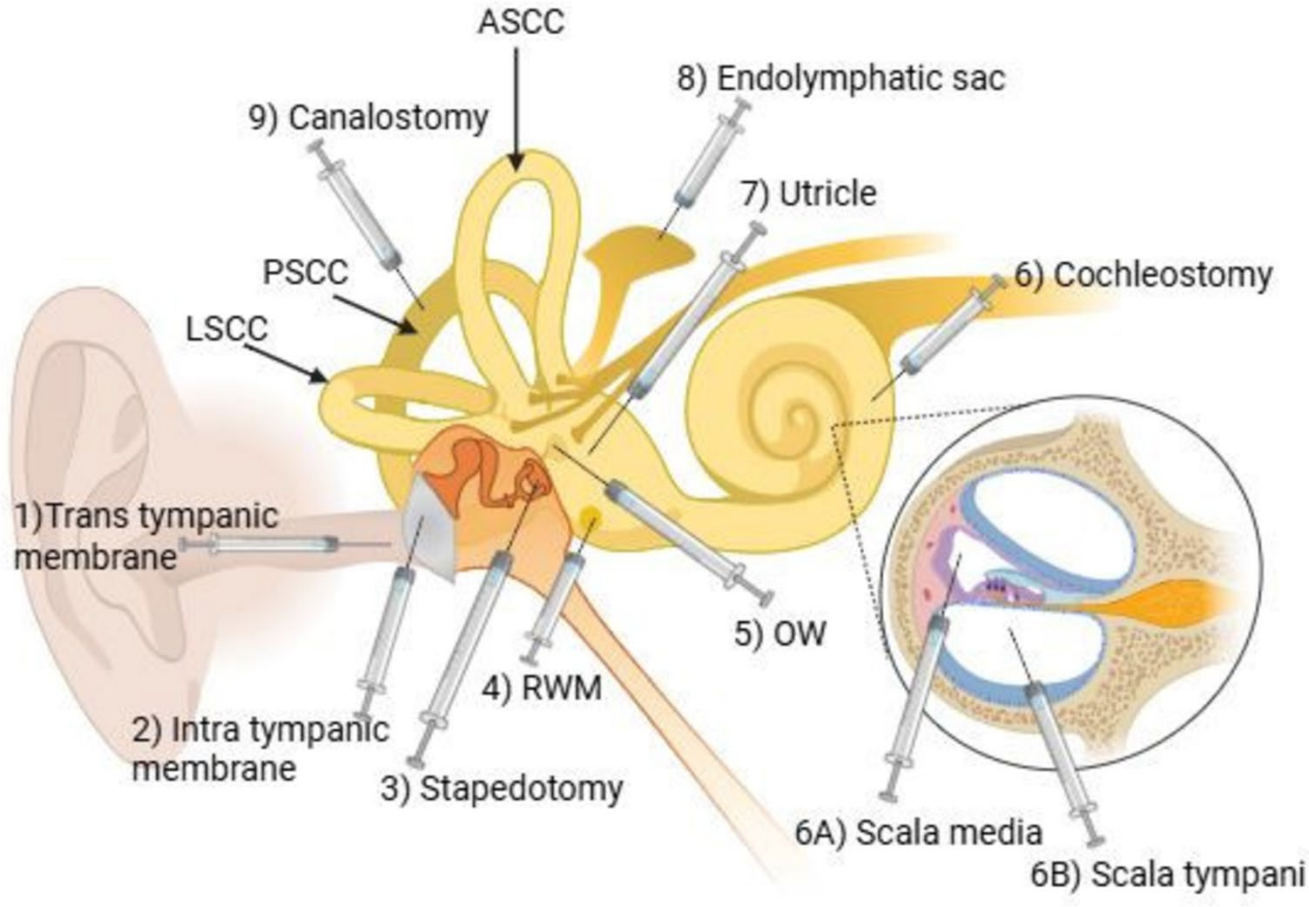
Diagram showing the different drug delivery routes. RWM: round window membrane, OW: oval window, TT: Transtympanic, PSCC: posterior semicircular canal, LSCC: lateral semicircular canal, ASCC: anterior semicircular canal. The figure was generated using the BioRender tool

Gene therapy can be delivered to the inner ear through various routes, as shown in Fig. 3. *Trans and intra-tympanic* routes demand the entrance of the vector into the middle ear cleft. This route is relatively less invasive with minimal risk of cell damage. *Stapedotomy* delivers the drug through the stapes. Injection of a therapeutic agent through the round window membrane (*RWM*) diffuses it into the perilymph. The oval window (*OW*) is another perilymphatic route used [24]. Injection directly through the *utricle* has shown good results. Injection through the *endolymphatic sac* has been adopted in several studies [25, 26]. *Cochleostomy* is considered a successful approach where injection is transferred through the scala tympani and/or scala media endolymph that diffuses to the vestibular endolymph [27]. Cochleostomy and RWM have similar abilities of transduction for inner hair cells, but cochleostomy is technically riskier than the RWM due to the possibility of surgical trauma [28, 29].

In *canalostomy*, injection is through a fenestration in the posterior semicircular canal (PSCC) [30, 31]. This guarantees a wider distribution through the labyrinth. Combining injection through RWM and canalostomy has shown high efficiency of inner hair cells (IHC) transduction in the cochlea [32].

### Treatment approaches

**Fig. 4:**
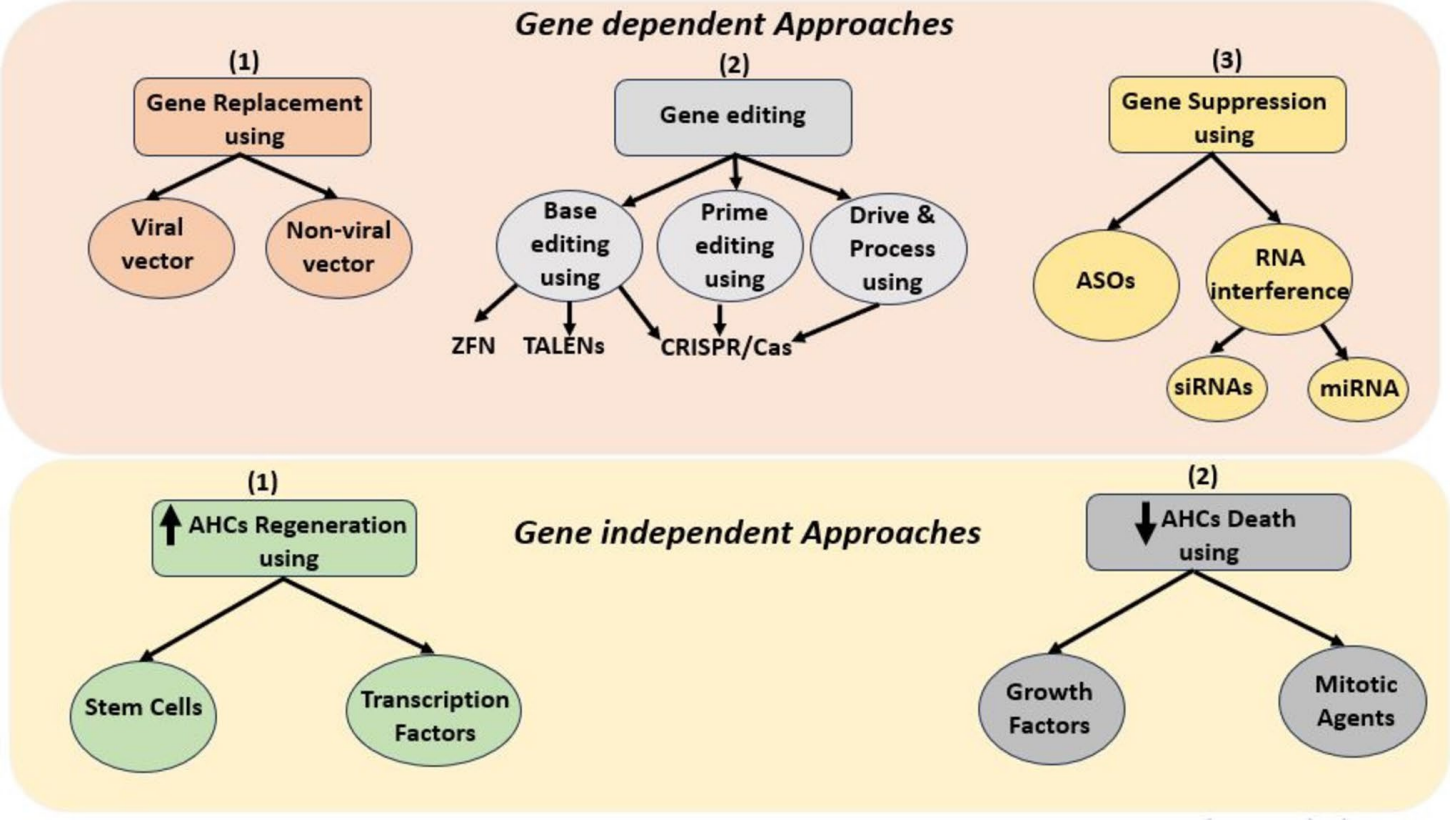
Diagram showing different gene-dependent and independent treatment approaches. ZFNs: zinc finger nucleases, TALENs: transcription activator-like effector nucleases, CRISPR/Cas: clustered regularly interspaced short palindromic repeats and CRISPR-associated protein, ASOs: antisense oligonucleotides, SiRNAs: short interfering RNAs, miRNAs: microRNAs, AHCs: auditory hair cells

Gene therapy trials were focusing on sensorineural hearing loss (SNHL). This section will discuss all therapy approaches used. A gene-dependent approach focuses on the introduction of a healthy copy of the defective gene or fixing the damaged copy. A gene-independent approach is concerned with increasing the number and efficiency of hair cells regardless of the causative gene defect, as shown in Fig. 4. Some representative animal model trials for each approach are tabulated in Table 3.

**Table 3:** Examples of studies using different gene therapy strategies

Strategy		Transfection tool	Route	Injection age	Gene target	Mouse model	Targeted cells	Result	Ref
**Gene Replacement**		AAV2/1	CO, RWM	P0-P3, P10-P12	*VGlut3*	*VGlut3* KO	IHCs	Normal ABR threshold (after 2 weeks), improvement in morphology of IHC ribbon synapse	[116]
		AAV-DJ, AAV-KP1	PSCC	P1	*TMPRSS3*	*TMPRSS3*^Tm1/tm1^	HCs, SCs	HL improvement (thresholds: 60 dB) for 4 months after treatment	[117]
		AAV9-PHP.B	RWM	P1	*PCDH15*	*PCDH15*-KO	HCs	HL improvement (thresholds: 40 dB) for 2 months after treatment	[118]
**Gene Suppression**	**RNAi**	Liposomes (siRNA)	RWM	P42-P45	*GJB2*	*GJB2* p. R75W	SCs	Hearing improvement to normal levels	[119]
		rAAV2/9	RWM	P0-P2	*TMC1*	*TMC1*-Bth	HCs	No progression for HL for 35 weeks after treatment	[120]
		AAV2/9	RWM with CS	P15-P90	*TMC1*	*TMC1*-Bth	HCs	No progression for HL for 12 weeks after treatment	[121]
	**ASOs**	Lipofectamine2000 (cationic liposome)	IP	P5, P10	*USH1C*	*USH1C* c.216G > A	HCs	HL improvement (thresholds:50 dB) for 3 months after treatment	[122]
**Gene Editing**	**CRISPR/CAS9**	AAV-PHP.eB	SM	P0-P3	*OTOF*	*OTOF* ^Q829X^	IHCs	Auditory function improvement reaching normal levels for 7 months	[123]
		AAV-PHP.eB	SM	P0-P2	*MYO6*	*MYO6* p.C442Y	HCs	HL improvement (thresholds:45 dB) for 5 months after treatment	[124]
**Non- gene dependent Approach**	**Maintenance and regeneration of AHCs**	Ad28.gfap.atoh1	PSCC	3 months	-	C57BL/6	HCs	Moderate recovery of hearing at 4 and 8 kHz after 2 months and development of auditory hair cells	[125]
		hESC-derived precursor cells (Stem cells approach)	CO	-	-	Pou4f3DTR/ +	HCs	Transplanted cells were viable and were able to differentiate into hair and supporting like cells	[126]
		slow-release gel with NT-3	RWM	24 h after exposure to noise	-	noise-exposed mice	HCs	Regeneration of pre- and post-synaptic elements at the hair cell / cochlear nerve interface coupled with a corresponding functional recovery	[64]

RNAi: interfering RNA, P:postnatal day, AHCs: Auditory hair cells, ASOs: Antisense Oligonucleotides, CO: Cochleostomy, RWM: Round Window Membrane, PSCC: posterior semicircular canal injection, CS: canal fenestration, IP: intraperitoneal injection, SM: Scala Media injection, HC: hair cells, IHC: Inner Hair Cells, SC: Supporting Cells, KO: Knock out, Bth: Beethoven mouse model, HL: hearing loss, hESC: human embryonic stem cells, dB: decibel

#### Gene replacement

Concept

Gene replacement is the most widely used approach. It is mainly used in loss-of-function biallelic recessive mutations and haploinsufficient dominant mutations [33]. It involves delivery of a functional copy of the gene (therapeutic transgene) in the form of complementary DNA (cDNA). AAV is the most widely used vector for escorting the therapeutic transgene.

Examples

A study used serotype8 AAV carrying the *VGLUT3* gene injected in postnatal mice at day 1 to 3 through PSCC showed hearing improvement with a 40 decibel (dB) threshold for up to 3 months after injection[34]. Another study was carried out using AAV to transport the *ESP8* gene to treat ARNSHL by injection through RWM for hair cell elongation in mice. The study concluded that the elongation of hair cells was proportional to the injected dose of the wild-type gene [35].

#### Gene suppression

Concept

It is used in negative dominance cases where the produced mutant protein can abolish the function of the normal protein produced by the other allele. Silencing can be accomplished on the transcriptional level or the translational level. This approach uses antisense oligonucleotides (ASOs) and short interfering RNAs (siRNAs) or microRNAs (miRNAs) [36, 37].

Examples

ASO-29 was used in the Ush1c^c.216G>A^ animal model that showed hearing improvement from 3 to 6 months after injection in several studies[38–40]. In a negative dominance SNHL involving the *GJB2* gene, the mutant allele expression was inhibited through placement of a resorbable gelatin sponge loaded with liposomes containing the siRNA specific for the mutated gene against the RWM. The mutant allele’s expression decreased to 30% [41].

#### Gene editing

Concept

Gene editing is concerned with endogenous gene disruption for gain-of-function mutations and correction for loss-of-function mutations [42, 43]. Unlike gene replacement, the length of the target gene is not a challenge since it needs only to deliver the gene editing tool of choice to the target cells. The most important tools are ZFNs (zinc finger nucleases) [44], TALENs (transcription activator-like effector nucleases) [45], and CRISPR/Cas9 (clustered regularly interspaced short palindromic repeats and CRISPR-associated protein 9) [46]. They are used to cut and modify DNA at one or several sites in the genome simultaneously. CRISPR-Cas9 has become the first choice due to the ease of designing guide RNA sequences for efficient targeted editing [47].

Examples

The CRISPR-Cas9 approach is used in cases of single-nucleotide substitution dominant hearing loss [48]. It was first applied to DFNA36, where it targeted the mutant *TMC1* allele in Beethoven mice, but low restoration of hearing was detected [49]. This was attributed to the lack of specificity of Cas9 to the mutant allele, which was later increased by introducing a proto-spacer-adjacent motif (PAM) variant of Cas9. This modified Cas9 helped prevent deafness in TMC1^Bth/WT^ mice for up to one year after injection, as well as preservation of normal hair bundle morphology in both IHCs and outer hair cells (OHCs) [50].

Updates of gene editing concept

The CRISPR/Cas9 system is in continuous modification to increase the specificity and selectivity to allow higher on-target and lower off-target results. This modification allowed prime editing[51, 52] instead of base editing, where more than one base can be edited in a short DNA sequence at the same time by recognizing and excising the defective part and replacing it with a newly synthesized part. Prime editing was able to excel base editing by performing some transversions through the interchange between purines and pyrimidines and avoiding deleterious double-strand breaks. A third and more comprehensive approach called Drive and Process (DAP) has been recently introduced [53] too, where multiplex base editing or prime editing can take place to repair a single base or a short DNA sequence simultaneously.

#### Gene-independent approach

Auditory hair cell regeneration

Regeneration of hair cells has been the main scope of this approach. Math1(Atoh1) is a positive regulator that helps in the differentiation of hair cells in the cochlear development stage. Some pre-clinical studies concluded that the overexpression of the Atoh1 triggers trans differentiation of cochlear supporting cells into hair cells using adenovirus as a carrier [54–56]. Atoh1 expression duration is regulated by upstream factors and co-activated by other downstream factors. This demands developing other approaches that take co-factors into consideration to guarantee reaching a normally functioning organ of Corti [57].

Stem cells could be differentiated into various cell types of the inner ear. This helped in protecting and repairing the sensory cells and neurons [58]. It uses mesenchymal stem cells (MSCs) derived from different sources, such as bone marrow and adipose-derived[59]. The transplanted MSCs reach the cochlea to be differentiated into hair and supporting cells, counteracting their loss. Guinea pigs, pigs, rats, and mice have been used in these studies [60–63].

Protective local treatments

Several studies focus on the transfer of genes encoding growth factors or mitotic agents to block the degeneration of inner ear neurons, allowing better performance outcomes from CI. Neurotrophin-3 (NT-3) prevents cochlear neuron degeneration [3]. It is important for the normal innervation of the cochlea, production, and maintenance of postnatal cochlear synapses. Delivery of NT-3 to the RWM of noise-exposed mice through a slow-release gel could protect cochlear synaptopathy by regeneration of synapses and preservation of nerve fibers [64].

### Gene therapy trials on humans

In the past few years, some pre-clinical gene therapy projects have been launched. Most of them are targeting some of the common hearing loss genes, common syndromes, or focusing on hair cell regeneration[65].

The first clinical trial on humans was launched in 2014, targeting 22 volunteers of an age range from 18 to 75 years with clinical trial ID (NCT02132130). The enrolled patients had unilateral or bilateral non-fluctuating severe to profound hearing loss. An Ad5 carrying the human transcription factor (Hath1) was used for hair cells regeneration. The results showed slight improvement.

A phase I/IIa study involving adult patients (15 patients in phase I and 44 patients in phase IIa) whose age ranged from 18 to 80 years, presenting with mild to moderate SNHL, has been carried out. The patients received an intratympanic gamma secretase inhibitor (GSI) that increases the expression of Atoh1 needed for hair cell differentiation. In phase I, the 15 patients were injected once, and in phase IIa, the patients were injected 3 times. No severe adverse effects were reported in phase I. However, in phase IIa, the average pure-tone threshold did not change from baseline through 6 and 12 weeks. The study concluded that the intratympanic delivery of the GSI is safe, but of no significant therapeutic results [66].

Special concern has been raised for targeting the *OTOF* gene, which causes auditory neuropathy spectrum disorder (ANSD) [67], as well as DFNB9, an ARNSHL. Several clinical trials aimed to provide the patients with correct copies of the *OTOF* gene.

One of these studies was carried out in China (ChiCTR2200063181), where six children were recruited with ages ranging from 1 to 18 years. They presented with severe to complete HL with no CI. All of them had confirmed biallelic *OTOF* mutations. All patients received a single injection of AAV1-hOTOF through RWM. Neither dose-limiting toxicity nor serious adverse events were reported through the 26-weeks follow-up period. Mild adverse effects, including increased lymphocyte counts and cholesterol levels, were observed. Five children had hearing recovery, shown by a 40–57 dB reduction in the auditory brain stem response (ABR) thresholds at 0.5–4.0 kHz with obvious speech perception improvement and ability to localize sound source [68, 69].

Another study depended on performing a single RWM injection of AAV-*OTOF* in two pediatric patients who presented with auditory neuropathy causing profound HL. A 5-year-old child received a unilateral injection as the other ear had CI, whereas an 8-years-old child received bilateral injections. The 5-years-old child showed improvement in the hearing threshold after one month, similar to that of the contralateral ear with CI. After three months, improvements in hearing were obtained at all frequencies, approaching normal levels without the hearing aid. The 8-year-old child who had bilateral injections also showed improvement in pure tone audiometry (PTA) and ABR. The study concluded that the AAV-*OTOF* injection represents a wider treatment window for DFNB9 [70].

It is observed that trials including children showed satisfactory restoration of hearing, allowing those children to acquire milestones as their peers. This might be attributed to neurodevelopmental windows for language acquisition and auditory plasticity. The first few years of life are important for first language acquisition, with high brain sensitivity for linguistic input and development of foundational language processing skills [71, 72].

## Challenges for gene therapy

### Inability to find an animal model coinciding with humans in all auditory aspects

In the past, zebrafish, birds, and rodents were used in vivo gene delivery experiments [73, 74]. However, rodents resemble the human inner ear structure [75, 76] but differ in the auditory development timeline [77]. The cochlea of the mouse reaches maturation 2–3 weeks after birth, whereas in humans, it is fully functioning at birth [78, 79]. The maturation of the human auditory system in the embryonic stages is affected by the proper expression of some genes, such as Atoh1 [80] and the Pax paired-homeobox gene family [81]. Their absence causes irreversible damage that can’t be restored by postnatal transgene introduction. To mimic neonatal mice therapy trials in humans, therapy should commence during gestation, which might expose the embryo to unstudied risks. Also, humans and mice differ in viral tropism, which could lead to non-reproducible results of gene therapy [82].

The involvement of mature mice is more challenging than that of neonatal mice due to the difficulty in reaching the cochlea as it becomes embedded in the temporal bone. Experiments using mature mice also showed low efficacy of transduction of the outer hair cells using viral vectors [31].

### Difference in causative genes, target cells, and optimum timing

Increasing genetic diversity represents a challenge for providing a tailored gene therapy. The optimal therapeutic intervention timing depends on the produced protein, time, and target cells [83]. Also, determining the most critical isoform of the gene to be replaced is another challenge [84, 85]. This makes it difficult to design a “one-size-fits-all” gene therapy, but in general, early childhood intervention is expected to show better outcomes compared to adulthood.

### The blood-labyrinth barrier (BLB)

BLB maintains ionic homeostasis, isolation from pathogens in the blood, and contributes to the semi-immune-privileged nature of the inner ear [25]. Lipopolysaccharide can increase its permeability, helping intracochlear therapeutic delivery [86, 87].

### The low cargo capacity of AAV

AAV has a low cargo capacity (4.8 kb) [88]. This is increased by using dual and triple-AAV. AAV delivery is dependent on the cell’s internal DNA repair pathway speed, which is relatively slow in the inner ear [89]. The increased viral genome load might lead to insertional mutagenesis, leading to cancer development [90]. This problem can be tackled through the addition of molecular therapies that enhance the DNA repair capacity of cells[91].

### PE and DAP can lead to unintended editing

Multiplex base editing or PE through the DAP approach might lead to unintended editing of nested genes, which are complete or parts of genes that reside in other genes. This might affect the functions of other unintended genes [82].

### Risk of structural damage during therapy administration

Hair cells might be injured due to induced pressure during injection, leading to loss of residual hearing [32, 92]. RWM route is relatively less invasive than cochleostomy, where injection of the drug is done through the existing round window membrane, whereas cochleostomy requires drilling an opening in the cochlea for the drug to be injected, which could be risky. This might justify that most of the recent studies favor RWM rather than cochleostomy in the procedure of CI [93–95] and in gene therapy[66, 69, 70]. Refining the delivery routes to reach an optimal choice with the least traumatic effect on inner ear structures will increase the total therapeutic output of gene therapy.

### The inner ear fluids-brain connection is a double-edged weapon

The brain and the inner ear fluids are connected through the cochlear aqueduct [96]. This represents a threat of distribution and integration of viral genetic material into other tissues[8] such as the brain, by escaping the cochlea[97]. Reaching blood can develop antibodies against the AAV or the loaded transgene [98]. This might cause an antigen–antibody reaction, leading to the destruction of gene therapy and a cytokine storm[82].

Primary safety data from one of the clinical trials showed detection of transgene DNA in a dose-dependent manner in the blood distribution on the first day after injection. The amount decreased to below the detection limit 4–8 days later. No abnormalities in any body system were reported before or after injection. No abnormal changes in hearing function related to the surgery were observed so far [70].

### Incomplete assessment of the durability of the positive effect

The long-term durability of positive effects hasn’t been guaranteed, as the follow-up of treated mice usually lasts for a few weeks. About 50% of the proof of concept studies reported a decline of the positive effects after 2 months of injection [24], which necessitates multiple doses due to the functional changes that occur in the inner ear hair cells over time, which also affects the long-term efficiency [99].

### Lack of reinnervation by spiral ganglion neurons

Some trials tried to tackle the degenerated hair cells problem through inducing mitotic regeneration, encouraging trans differentiation of nearby supporting cells into hair cells [55, 56], which were rendered nonfunctional due to a lack of re-innervation by the spiral ganglion neurons[100].

### Ethical considerations

In utero gene therapy has been a controversial issue. A meticulous risk/benefit ratio should be considered, as it holds a promise of preventing or treating diseases, but raises ethical and safety concerns due to worries about the emergence of new diseases and adverse health effects in future generations[101]. A long-term commitment to follow up should be added to the consents to guarantee a long-term safety monitor. However, some opinions favor using gene therapy, despite the risks that might be induced during the amniocentesis process, for embryos in cases of lethal diseases that might lead to termination of pregnancy or neonatal death[102].

### Cost and access barrier

The cost of developing a gene therapy is a real challenge owing to the therapy’s complexity, good manufacturing practice, and clinical trials [103]. The ear is not a closed system, which might require multiple doses of the drug. On the contrary, the retina is a closed system that needs small amounts of drug for treatment and hence relatively lower cost [104]. Gene therapy for Spinal Muscular Atrophy using Zolgensma is another example of a one-time gene therapy offering significant survival benefits for patients [105]. This renders the cost of HL gene therapy a problem that needs to be tackled.

## Conclusion

Hearing loss is a worldwide health problem. Early screening and immediate intervention through hearing aids or CI could help in achieving better outcomes. However, it is a good model for gene therapy due to the dominance of monogenic defects. The use of gene therapy approaches alone or in combination with CI can give hope for near-normal hearing. A large number of studies highlight the importance of molecular diagnosis and gene therapy as a valid treatment option for hereditary deafness, with successful outcomes detected. However, challenges remain in terms of delivery methods, long-term efficacy, and safety. Still, a lot of research should be done to nick the gaps and enhance the benefits of this approach.

## Data Availability

No datasets were generated or analysed during the current study.
